# Inhibitory effects of *Cheongsangbangpoong-tang* on both inflammatory acne lesions and facial heat in patients with acne vulgaris: A randomized controlled trial protocol

**DOI:** 10.1186/s12906-016-1000-9

**Published:** 2016-01-22

**Authors:** Kyuseok Kim, Kwan-Il Kim, Junhee Lee

**Affiliations:** 1 Department of Ophthalmology & Otolaryngology & Dermatology, College of Korean Medicine, Kyung Hee University, 23 Kyungheedae-ro, Dongdaemun-gu Seoul, 02447 Republic of Korea; 2 Department of Sasang Constitutional Medicine, College of Korean Medicine, Kyung Hee University, 23 Kyungheedae-ro, Dongdaemun-gu Seoul, 02447 Republic of Korea; 3 Korean Medicine Clinical Trial Center, Kyung Hee University Korean Medicine Hospital, 23 Kyungheedae-ro, Dongdaemun-gu Seoul, 02447 Republic of Korea

**Keywords:** Acne vulgaris, Inflammatory lesion, Facial heat, Herbal medicine, *Cheongsangbangpoong-tang*

## Abstract

**Background:**

Due to increasing interest from acne patients concerned about the side effects associated with conventional therapies, complementary and alternative medicine (CAM) has been suggested as a new therapeutic modality for acne vulgaris. Herbal medicine is one of these CAM treatments. *Cheongsangbangpoong-tang* (CBT) is a common herbal formula used in patients with acne vulgaris in the clinical practice of Korean Medicine (KM). However, despite the common use of CBT in clinical practice, the current level of evidence is insufficient to support an inhibitory effect of CBT on inflammatory acne lesions and facial heat. Therefore, this study was designed to assess the inhibitory effect of CBT on both inflammatory acne lesions and facial heat.

**Methods/design:**

A randomized, double-blind, parallel-group, and placebo-controlled trial will be conducted. Fifty-six participants with acne vulgaris will be randomized into one of two groups: the CBT or placebo groups. After randomization, participants will be prescribed either CBT or placebo three times a day at a dose of 5 g after meals for 8 weeks. The following outcome measurements will be used in the examination of subjects: the mean percentage change and the count change of the inflammatory and non-inflammatory acne lesions, the temperature of facial points on digital infrared thermal imaging (DITI), serum cortisol, serum dehydroepiandrosterone-sulfate (DHEA-S), visual analogue scale (VAS), investigator global assessment (IGA), and severity score on the Korean Acne Grading System (KAGS) from baseline to the end of the trial.

**Discussion:**

This trial will provide evidence regarding the inhibitory effect of CBT on inflammatory acne lesions and facial heat. The findings of this trial may have important implications for the more widespread use of CBT for the treatment of acne vulgaris.

**Trial registration:**

The trial is registered with the Clinical Research Information Service (CRiS), Republic of Korea: KCT0001468.

## Background

Acne is a multifactorial inflammatory skin disease of the pilosebaceous unit [1]. With respect to pattern identification, which is defined as “the process of overall analysis of clinical data to determine the location, cause and nature of a patient’s disease” [2], acne is deeply relevant to the ‘*Heat*’ pattern [3, 4]. Patients diagnosed as suffering from a ‘*Heat*’ pattern, such as the ‘*Wind-Heat*’ or ‘*Dampness-Heat*’ patterns, in their dermatologic disease have characteristics similar to the inflammatory state [5].

In Korea, *Cheongsangbangpoong-tang* (CBT) has been approved by the Korea Food and Drug Administration as a prescription for clinical use in patients with acne, and is most frequently used by Korean Medicine (KM) clinicians in patients with acne vulgaris. CBT was first mentioned in ‘*Wanbinghuichun’* as a novel herbal therapeutic formula. It cleans the *upper energizer pathogenic heat*, which is considered the major pathogenic cause of acne in Korean traditional medicine, and treats the sores and furuncles on the head and face [6]. However, until now, no well-designed and randomized clinical trial supporting this classic substance has been performed.

We hypothesized that CBT inhibits acne inflammatory lesions and facial heat mainly displayed by patients identified as suffering from a ‘*Heat*’ pattern. The purpose of this research is to evaluate the inhibitory effects of CBT, an herbal formula, on both inflammatory acne lesions and facial heat in patients with acne vulgaris.

## Method/design

### Aim of the study

The primary objective of the present trial is to investigate the inhibitory effects of the herbal formula CBT on both inflammatory acne lesions and facial heat in patients with acne vulgaris.

The primary null hypothesis is as follows: CBT treatment is superior to placebo treatment in its inhibitory effect on the mean percent change in inflammatory acne lesions between baseline and the end of the trial.

### Design

This study is a randomized, placebo-controlled, parallel-group, double-blind and single-center trial. This trial is registered with the Clinical Research Information Service (CRiS), Republic of Korea: KCT0001468. The study will be sequentially conducted as follows: enrollment after screening via inclusion and exclusion criteria, randomization, a treatment period of 8 weeks, and assessment.

**Fig. 1:**
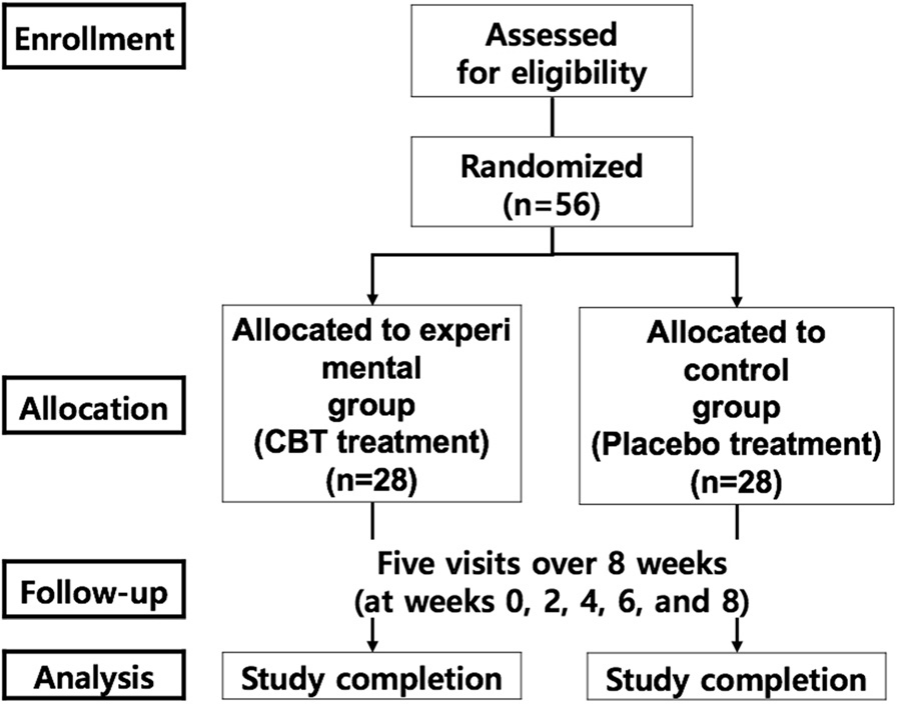
Flow chart of the study

In addition, the protocol will be conducted in accordance with the Declaration of Helsinki and the Good Clinical Practice Guidelines [7]. All investigators are appropriately qualified to conduct and supervise the trial. All patients will provide written informed consent prior to study entry. The flow chart of this study is shown in Fig. 1.

### Ethics

The study protocol and written informed consent were approved by the Institutional Review Board (IRB) of the Kyung Hee University Korean Medicine Hospital (KOMCIRB-150213-HR-006). Each participant will be provided with information regarding the study protocol. Written informed consent will be obtained from each patient. The privacy of all participants will be protected. Personal medical records will be reviewed by investigators, who will promise to keep the content confidential. Data anonymity will be used in the process of data management. Finally, the data of all subjects will be kept in the document storage room of Kyung Hee Korean Medicine-Clinical Trial Center (K-CTC).

### Participants and eligibility

#### Inclusion criteria

Patients with acne vulgaris aged over 19 years, who have more than 10 inflammatory acne lesions on their face (from the jawline margin to the front margin of hairline), and who agree to use only the intervention in this study for acne vulgaris treatment during this trial period are eligible to participate in this trial.

#### Exclusion criteria

We will exclude patients with laboratory findings in the following ranges: aspartate transaminase (AST) ≥ 60 IU/L, alanine transaminase (ALT) ≥ 60 IU/L, and creatinine > 1.2 mg/dl. Participants will be ineligible if they have undergone Western medical treatment or traditional medical treatment or taken oral contraceptives/antibiotics for acne within 2 weeks prior to recruitment or are pregnant, lactating, or planning to become pregnant during the study. Participants will also be excluded if they are not willing to comply with this study protocol. During the 8 weeks after enrollment in this trial, no concomitant acne therapy will be permitted.

### Recruitment

Participants will be recruited via an internet advertisement posted on the website of the Kyung Hee Medical Center and via posted flyers. A total of fifty-six patients will be recruited in this trial.

### Randomization and allocation concealment

Using a stratified block randomization method, 56 eligible patients will be randomly allocated to the CBT or placebo groups in a 1:1 ratio in blocks of 4. Random numbers will be generated by a computerized random-number generator using the block-randomization method of a software program (R 3.2.2) for sequence generation by an independent statistician. The randomization lists will be concealed in a lightproof sealed envelope. The sealed envelopes will be kept by the independent statistician of the study. Participants, investigators and outcome assessors will be blinded to the treatment allocation throughout the course of the study.

### Blinding and code breaking

Drugs (CBT and placebo) have been coded, providing masking to the treatment assignment to ensure double blinding. Patients, clinicians, outcome assessors, and investigators are unaware of treatment group assignments or block size. If a participant has a serious adverse event and immediate cessation of trial medication is required, the blinding code will be broken.

### Intervention

#### CBT and placebo groups

CBT granule is approved by the Korea Food and Drug Administration as a prescription for use in clinical practice for patients with acne. The components of CBT are presented in Table 1. Each of the CBT crude materials was extracted with 500 mL of hot water for 48-72 hours. The extracts were filtered, concentrated, lyophilized, and stored at -60 °C. The yield of CBT dried extract was approximately 15.8 % (w/w, dry weight 1.5 g). The other excipients (corn starch and lactose CARB) were added to this dried CBT extract. CBT granules were purchased from the Han Kook Sin Yak Pharm. Co., which is equipped with a Korean good manufacturing practice (KGMP) facility. A voucher specimen (# KCTC 001502) was deposited at K-CTC.

**Table 1:** Components of *Cheongsangbangpoong-tang* (CBT) granules (5 g / dose)

Scientific name	Amount (g)
*Schizonepeta tenuifolia*	0.5
*Coptis japonica Makino*	0.5
*Mentha arvensis var. piperascens*	0.5
*Ponciri Fructus Immaturus*	0.5
*Glycyrrhiza uralensis FISCH*	0.5
*Gardenia augusta*	1.0
*Cnidium officinale*	1.0
*Scutellaria baicalensis*	1.0
*Forsythia koreana*	1.0
*Angelica dahurica*	1.0
*Platycodon grandiflorum*	1.0
*Ledebouriella seseloides*	1.0
*Corn starch*	1.2
*Lactose hydrate*	2.3
Total	5.0

Participants assigned to the placebo group will receive a placebo prescription instead of the herbal formula CBT throughout the 8-week post-randomization period. The placebo will not have any clinical or pharmacological effects. The placebo is almost identical to the test drug in appearance, smell and taste. This placebo preparation will be produced and purchased from the Jeonnam Biofood Technology Center, which is equipped with a Korean good manufacturing practice (KGMP) facility. The placebo granules, designed to match the CBT granules, to be used in this trial are identically sized granules filled with lactose powder (95.2 %), dextrin (2.94 %), herbal incense (0.7 %) and coloring (food coloring such as brown food coloring) (1.16 %), without any markings or active ingredients.

All drugs are concealed in uniform packages with the same labels in the Jeonnam Biofood Technology Center and each package contains a 28-day supply. Patients in the CBT or placebo groups will take each packaged granule containing either 5 g CBT or 5 g placebo as a single dose. All drugs will be dissolved in 200 ml warm water and will be taken orally after each meal 3 times daily for 8 weeks. During the study, patients will be visited three times by the investigators. We will establish the window for visits as ±3 days. Details of the study procedures are shown in Table 2.

**Table 2:** Detailed study procedures

Items	Screening/run-in period	Visit 1 (baseline)	Visit 2	Visit 4 (endpoint)
Week of the trial	-1	0	4	8
Demographic information-taking	+			
Informed consent form	+			
General medical history-taking	+			
Photographing the patient’s face Count of inflammatory/non-inflammatory lesions	+	+	+	+
Blood tests (AST, ALT, creatinine, serum cortisol, serum DHEA-S)	+			+
Urine pregnancy test	+		+	+
Digital infrared thermal imaging		+	+	+
Randomization & allocation	+			
Distribution of treatment or placebo		+	+	
Visual analogue scale		+	+	+
Investigator Global Assessments		+	+	+
Korean Acne Grading System		+	+	+
Pattern identification for acne	+			
Monitoring adverse events			+	+
Compliance check			+	+
Blinding check				+

### Outcome measures

#### Primary outcome

Primary outcome will be presented as the mean percentage change of inflammatory lesion counts from baseline to the end of the trial [8, 9].

### Secondary outcomes

Secondary outcomes will evaluate the mean percentage change of non-inflammatory lesions and the total of both inflammatory and non-inflammatory lesions, the differences in temperatures of the DITI points on the face, Visual Analogue Scale (VAS) [score 0 (no symptoms) to 100 (severe symptoms)], and the Investigator Global Assessment scale for Facial Acne Vulgaris (IGA; after evaluation from score 0 (clear) to 4 (severe)) [10] from baseline to the end of the trial (at 8 weeks) between CBT treatment and the placebo control groups. Serum cortisol and dehydroepiandrosterone-sulfate (DHEA-S) will be assessed using clinical laboratory tests at weeks 0 (baseline) and 8 (end of the trial).

VAS and IGA will be checked every session from baseline (just before randomization) through the 8 weeks after randomization. A Lumix DMC-LX2 digital camera (Panasonic, Osaka, Japan) will be used to photograph the front and both sides of each patients face, so that the inflammatory and non-inflammatory lesions can be counted independently at weeks 0 (baseline), 4 and 8 (end of the trial) by a clinical specialist who has been in clinical practice for more 5 years. We will consider black and white head comedones to be non-inflammatory lesions and the inflammatory papules, pustules and cysts to be inflammatory lesions.

Correlations between the inflammatory lesion count and the temperatures of the DITI points at baseline, 4 weeks, and 8 weeks, and the frequency of abnormal liver and kidney function during this trial will also be secondary outcomes. Additionally, we will analyze the correlation between the temperatures of the DITI points and the severity of the Korean Acne Grading System (KAGS) at baseline and the end of the trial (at 8 weeks). Finally, we will analyze the success rate of blinding between the actual treatment and the perceived treatment allocations.

### Withdrawal, dropout, discontinuation and compliance

Participants will be allowed to withdraw at any time during this clinical trial. Participants who wish to withdraw will be offered the option to cease trial intervention, but continue the visits for outcome measurement. Participants who withdraw will be followed to investigate the reason for withdrawal. Participants may be advised to discontinue this trial if there is a serious intervention-related adverse event or if the participant is noncompliant with the study procedure. CBT or placebo remaining after each session will be quantified in order to measure and enhance medication compliance. Participants whose compliance with CBT or placebo is ≤ 80 % of the total will be considered to have dropped out.

### Standard operation procedures for quality assurance

We have established detailed standard operating procedures for this clinical trial and have educated all practitioners and set qualification standards to make sure the patients are treated with high standards and in accordance with the trial protocol. Practitioners and medical equipment managers attended a 2-day training workshop about the trial procedures and were educated on methods for taking a picture of the patient’s face and how to examine the DITI, serum cortisol, DHEA-S, VAS, KAGS and IGA.

A written protocol and standardized recording documents will be provided. All practitioners and outcome assessors will be blinded to group allocations, and medical equipment managers will not participate in the interventions.

### Adverse events and safety monitoring

All unexpected adverse events related to CBT or the placebo intervention will be reported to the investigator by participants and written on the individual case report form by the investigator. Safety will be assessed by the reporting of clinical laboratory tests and adverse events. Clinical laboratory tests, including AST / ALT and creatinine, will be performed at screening and week 8 (the end of the trial). Each participant will be monitored for adverse events (pain at acne lesions or other sites, nausea/vomiting, fatigue, allergic reaction, and any adverse events related to intervention) after each visit.

### Statistical methods

#### Statistical analysis plan

Statistical analyses will be conducted on both intension-to-treat (ITT) and per-protocol (PP) bases, with a 95 % confidence interval using SPSS version 12.0 for Windows. Based on the ITT population, the primary outcome and safety outcome will be tested. Secondary outcome will be analyzed on both the ITT and PP populations. The ITT analysis will consider all randomized participants with at least one measurable outcome value during the study. Missing values from drop-out participants will be imputed by the last observation carried forward (LOCF) method. Data will be displayed as the mean ± standard deviation (SD) for continuous data or n (%) for categorical data.

### Baseline data and outcome data

An independent *T* test will be performed for comparison of the baseline values between the CBT and placebo groups. The independent *T* test will be used to examine the mean percentage change of the inflammatory/non-inflammatory lesion counts, the differences in the temperatures of the DITI points on the face, sebum level, serum cortisol, DHEA-S, VAS, and IGA at baseline and at the end of the trial (at week 8) between the CBT treatment and placebo control groups. Safety tests (AST, ALT and creatinine) will be analyzed using the independent *T* test at screening and at the end of the trial (at week 8) between the CBT treatment and placebo control groups. A repeated measured analysis of covariance (ANCOVA) test will be used to examine the percentage change of inflammatory and non-inflammatory lesions, differences in the temperatures of the DITI points on the face, VAS, and IGA at weeks 0, 4, and 8 when controlling for baseline and other covariates. The correlation between inflammatory lesion count and the facial skin superficial temperature points at baseline, 4 weeks, and 8 weeks will be examined using the Pearson correlation test. Additionally, we will analyze the correlation between the temperatures of the DITI points and the severity of the KAGS using the Pearson correlation test. The change of KAGS from baseline to week 8 will be assessed using a Chi-square test or Fisher’s exact test. All adverse events reported during the study will be included in the case report forms; the incidence of adverse events will be calculated. The percentage of subjects with adverse events in each group will be calculated and compared using a Chi-square test or Fisher’s exact test. The evaluation of blinding success between the actual treatment and the perceived treatment allocations will be performed using a Chi-square test or Fisher’s exact test.

### Sample size

Calculation of the sample size for this trial was based on the effect size of the primary outcome, mean percent change of inflammatory acne lesion counts from baseline to week 8, reported in a previously published study [11]. The mean and standard deviation (SD) of the mean percent change of inflammatory acne lesion counts were -32.4 ± 44 and -15.1 ± 44.9 for the active treatment and control groups, respectively. To achieve 80 % power (β = 0.20), a significance level of 0.05 (α) in a two-tailed test, calculated using Cohen's d effect size values for t-tests (0.39), 28 participants, based on an estimated 20 % dropout rate, are needed for each group. This results in a required total of 56 for the trial. The mean and SD of the mean percent change of inflammatory acne lesion counts from this pilot study will be used for recalculation of the sample size for larger trials.

## Discussion

CBT is widely used for the treatment of acne in clinical practice, but the underlying mechanism has not yet been proven. Moreover, there has been only limited research on its efficacy for inflammatory acne lesions or facial heat sensitivity. Thus, this clinical research trial was designed to provide evidence of the inhibitory effect of CBT on inflammatory acne lesions or facial heat sensitivity in patients with acne.

Our research was designed to be precise, in that we will use the placebo to maintain the double blinding of all assessors and practitioners, and have provided prior education to all practitioners. Blinding is an important scientific method in randomized controlled trials (RCT) [12]. Most RCT intend to include a double-blind design to exclude the potential influence of the natural course of the disease or any expectations of the participants and investigators [13]. In reality, because of the unique sensory and macroscopic characteristics, such as the appearance, taste and smell of herbal preparations, it is extremely difficult to prepare a placebo that is perceived to be identical to the real herbal formula while lacking any pharmacological or toxic activities [12, 13]. Consequently, we will perform an evaluation of blinding success.

The trial duration of 8 weeks was determined after considering the following issues: the lasting effectiveness time of the intervention and its tolerability by and compliance of the participants. This protocol was developed according to the guidelines for randomized controlled trials investigating Chinese herbal medicine [14] and follows the nine CONSORT checklist items for RCTs of herbal medicines [15]. We considered the main elements for conducting a high-quality RCT, such as randomization, sequence generation, allocation concealment, blinding methods, proper sample size, compliance with the protocol, and adequate outcome measures [15]. This research will provide important evidence regarding the more widespread use of CBT for the treatment of acne vulgaris.

### Trial status

Participant recruitment will commence on October 19, 2015. It is anticipated that the trial will be completed in April 2016.
